# Markers of Field Cancerization: Proposed Clinical Applications in Prostate Biopsies

**DOI:** 10.1155/2012/302894

**Published:** 2012-05-14

**Authors:** Kristina A. Trujillo, Anna C. Jones, Jeffrey K. Griffith, Marco Bisoffi

**Affiliations:** ^1^Department of Biochemistry and Molecular Biology, University of New Mexico Health Sciences Center, Albuquerque, NM 87131, USA; ^2^New Mexico Cancer Center, University of New Mexico Health Sciences Center, Albuquerque, NM 87131, USA

## Abstract

Field cancerization denotes the occurrence of genetic, epigenetic, and biochemical aberrations in structurally intact cells in histologically normal tissues adjacent to cancerous lesions. This paper tabulates markers of prostate field cancerization known to date and discusses their potential clinical value in the analysis of prostate biopsies, including diagnosis, monitoring progression during active surveillance, and assessing efficacy of presurgical neoadjuvant and focal therapeutic interventions.

## 1. Introduction

### 1.1. Definitions of Field Cancerization

The term “field cancerization” or “field effect” was originally introduced by Dr. Slaughter and colleagues in 1953 in the context of oral squamous cell carcinoma [1]. It was used to describe the presence of histologically abnormal tissue surrounding primary cancerous lesions and was proposed to be the reason for the occurrence of multifocal tumors and for the development of locally recurrent cancer. Field cancerization was much later proposed for other organ systems, including prostate [2–7]. While its original clinical implication remained the same, that is, the occurrence of multifocality and cancer recurrence, it must be emphasized that the definition of field cancerization has changed over time. Of note, due to the tremendous progress in molecular biology and biotechnology since the 1950s, the description of field cancerization has changed from a largely histological to a more refined molecular perspective. This change is perhaps best reflected in the following definitions reported in the literature. Accordingly, the original intention by Slaughter and colleagues [1] was to describe: *“The presence of histologically abnormal tissue surrounding cancerous lesions.”* Höckel and Dornhöfer extended this definition by using the term “hydra phenomenon of cancer” [8]: *“The monoclonal or multiclonal displacement of normal epithelium by a genetically altered but microscopically undistinguishable homologue.”* In addition to the transition from a purely histological to a molecular description, another notable change is the introduction of the concept of histological normalcy as part of tissue pre-malignancy. Indeed, this newer definition is now established for organs developing solid tumors, including prostate cancer, and denotes the occurrence of molecular alterations in structurally intact cells that are part of histologically normal tissues.

Several aspects of prostate field cancerization remain unanswered. An immediate question is whether the “field” of molecular alterations is exclusively of precursor nature, or whether it is induced by the tumor. This is further complicated by the fact that this influence could affect all types of cells, including cells of the stroma. The latter has indeed been discussed as “reactive stroma”, which develops to support, or even induce oncogenesis and cancer progression [9, 10]. This heterotypic view has recently been proposed in the context of the tissue organization field theory as opposed to the well-established somatic mutation theory [11].

### 1.2. Prostate Field Cancerization and Multifocality

Prostate cancer tends to present as multifocal disease with reported rates of up to 90% of prostates containing two or more cancerous foci at the time of clinical diagnosis [12]. Multifocality is viewed as a major contributor to the complexity and inaccuracy of all aspects of prostate cancer clinical assessment and management [12–15]. For example, it greatly complicates staging and grading because individual foci usually display extensive heterogeneity and thus could progress at different rates depending on the nature and extent of their genetic instability and their microenvironment. Fortunately, some of these challenges can be partially counteracted by sophisticated histological assessments, for example, the Gleason sum score which combines the first most common with the second most common grade of dedifferentiation, enhances the accuracy of prognosis and patient outcome and consequently defines the most optimal treatment modality [16].

Several mechanisms underlying the development of multifocality can be envisioned. Accordingly, different cancerous foci could evolve truly independently from each other; they can remain isolated or fuse if they are in close proximity to each other. The latter would explain the existence of different grades of dedifferentiation often found within one lesion, for example, the concomitant occurrence of the cancer precursor prostatic intraepithelial neoplasia (PIN) with cancer of different Gleason grades [6, 17]. Alternatively, multifocality could be explained by intraductal migratory cells undergoing dysplasia; in this case, intrafocal heterogeneity could be due to concomitant development of different genetic aberrations of different cell clones of an individual lesion. It is not inconceivable that prostate cancer multifocality could be due to a combination of these mechanisms occurring concomitantly. Regardless of the mechanisms that lead to prostate cancer multifocality, it is compatible with the concept of field cancerization. In fact, a field effect, defined as an underlying “predisposition” in areas of the prostate gland, has been proposed to play a causal role in the development of multifocal lesions [6, 12].

## 2. Field Cancerization: Possible Applications in Prostate Biopsies

The necessity to recover tissue from the prostate gland by transrectal or transperitoneal biopsy can be indicated for several reasons, including confirmatory diagnosis, monitoring progression during active surveillance, and assessing efficacy of presurgical neoadjuvant and focal therapeutic interventions. In this paper, we will make a case for the potential clinical value of field cancerization to several applications involving the use of biopsies. In particular, we will discuss the possibility that specific markers of field cancerization could be useful and complementary biomarkers for the accurate clinical assessment of prostate biopsy tissues. At least three excellent recent reviews have provided lists of molecular factors that are indicative of field cancerization in human prostate tissues [3–5]. Although research on field cancerization in prostate tissue is relatively new compared to other organ systems, including head and neck as well as breast [18, 19], this list has become quite impressive due to the increasing acceptance of this concept. To fully appreciate the diversity of markers of prostate field cancerization, we provide here an updated overview, inclusive of stromal markers that fit the definition of field cancerization (Table 1). This list emphasizes at least two important insights into the biology of field cancerization in prostate tissues: first, field cancerization is manifested at all levels of the biological information flow and molecular regulation, that is, at the genetic, epigenetic, transcriptional, and posttranscriptional level. Second, field cancerization encompasses several cellular processes, including proliferation, metabolism, inflammation, DNA repair, and stromal/epithelial interactions. In theory, these broad biological characteristics of prostate field cancerization should increase the potential clinical value of its markers, especially when used in combination with other clinically established indicators.

The clinical use, as specifically applied to prostate biopsies that could greatly benefit from well-characterized markers of field cancerization are discussed in the following sections. The markers of prostate field cancerization listed in Table 1 were extracted from the literature published in the PubMed database of the US National Library of Medicine of the National Institutes of Health (http://www.ncbi.nlm.nih.gov/pubmed/). The following key words were used in multiple combinations: “(bio)markers, prostate, biopsy, histologically normal, adjacent, field cancerization, field effect, diagnosis, prognosis, margins, active surveillance, neo-adjuvant therapy, and focal therapy.” Of note, the focused analysis of tissue adjacent to tumors in general is rare, as it is most often used as a mere control for analyses specific to the tumors [20]. Furthermore, the use of the terms “field cancerization” or “field effect” is new in prostate cancer research. Therefore, it is expected that some reports addressing such analyses may be missing herein. However, rather than providing a complete list of possible markers of field cancerization and details about their findings, we emphasize in this paper their potential as biomarkers in prostate biopsies.

### 2.1. Prostate Field Cancerization and Diagnosis

**Figure 1:**
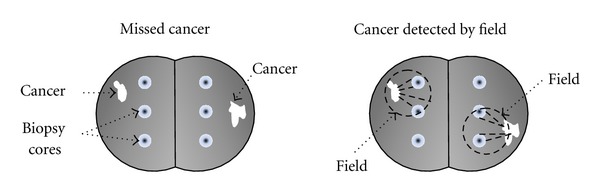
Improved diagnosis of prostate cancer by avoiding false negative biopsies through the use of markers of field cancerization. Biopsy cores (small circles) miss the two small cancer foci (white irregular structures; left); the field associated with the cancer foci (dashed circles) is detected by the biopsies (right).

The risk of having prostate cancer is currently assessed by the triad of elevated serum prostate specific antigen (PSA; typically ≥3 ng/mL), abnormal digital rectal examination (DRE), and transrectal ultrasonography (TRUS) [21]. However, these indicators are *per se* not sufficient for the accurate diagnosis of cancer because of low sensitivity (especially for DRE and TRUS) and specificity (especially for PSA) [22, 23]. Rather, they justify the patient's referral to the removal of biopsy cores for confirmatory diagnosis of cancer by histological assessment [21, 24]. Despite substantial controversy (further discussed in Section 2.2), PSA and DRE are often used to screen for prostate cancer leading to millions of analyzed needle biopsies each year in the USA [21, 24, 25]. A major problem inherent to biopsies is that 30–50% of men with subsequently confirmed prostate cancer experience an initial false-negative diagnosis [25–27]. This is because biopsy core needles can easily miss smaller and inconspicuous lesions (Figure 1). Conceptually, it seems logical that this problem relates to the number of biopsies removed. However, the ideal number of biopsies necessary to accurately detect one or more cancer foci has been a controversial issue and has changed in the modern biopsy era. Under ultrasound guidance, 6 total cores were first recommended, three from each side of the prostate gland; this was termed “sextant biopsy.” In the late 1990s, this recommendation was changed to 10–12 cores taken from the mid and lateral peripheral zones because it was shown to increase cancer detection [28, 29]. However, since the problem of a high false-negative rate persisted, the concept of “saturation biopsy” was introduced thereafter [28, 30]. Saturation biopsy involves removing 20 to 40 cores, and its value for cancer detection is being investigated as both an initial procedure and as a secondary intervention (with a focus on lateral and apical cores; also see Section 2.4) for patients with negative initial biopsy but persistent elevated serum PSA levels. These patients are by definition at a higher risk of having cancer [28]. Regardless of the number of needle cores removed, a false-negative finding, either at first or subsequent biopsy, represents an important problem because it generates anxiety for the patient and represents a dilemma for the physician as to whether and what kind of further action is required, especially in the presence of persistently high PSA [27–29]. Furthermore, an increased number of biopsy cores present the possibility of more complications and discomfort for the patient.

**Figure 2:**
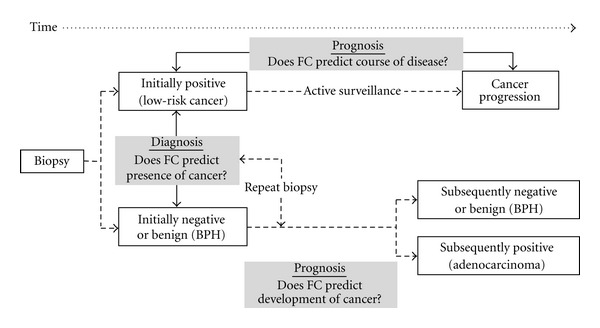
Nested case-control study design to investigate the diagnostic and prognostic potential of markers of field cancerization (FC) at the time of biopsy. Discrimination between positive and negative (or affected by benign prostatic hyperplasia, BPH) initial or repeat biopsies tests whether FC predicts the presence of cancer (left grey box). Discrimination between subsequently negative and positive biopsies after initial or repeat negative biopsies tests whether FC predicts development of cancer (bottom grey box). Prediction of cancer progression in initially positive biopsies indicating low-risk cancer during active surveillance tests whether FC is prognostic (top grey box).

The biological nature of field cancerization offers a potential means towards an improvement of this problem. The occurrence of molecular alterations that are associated with the presence of cancerous lesions, but that are not necessarily located in the same tissue area, potentially increases the target region of interest that can provide clinically meaningful information (Figure 1). This statement applies to both the traditional sextant biopsy, typically performed in the parasagittal plane between the lateral border and midline on both the right and left sides of the prostate gland as well as to the extended mode with cores removed more laterally in the anterior horns of the peripheral zone [30]. Depending on the nature of the field, that is, its extent and intensity, and on the discriminatory power of the marker(s) under investigation, it is not inconceivable that the necessity to detect histologically abnormal tissue or cells could become less important, especially when such markers are used in combination with other disease indicators such as PSA. The diagnostic value of markers of field cancerization is ideally tested in nested case-control studies, either prospectively or retrospectively. In this design, cases and controls are defined as patients with initially positive and negative biopsies, respectively. Retrospective studies offer many advantages with respect to the control of confounding factors: (i) patients derived from the same cohort can, and should be age-matched to control for potentially age related effects; (ii) the date of biopsy removal for cases and controls can be matched to account for changes in tissue quality over time; (iii) controls who remained free of cancer can be matched with cases who were at risk during the same time to assure the equal likelihood of cancer detection, which can be greatly confounded by many variables after an initial negative biopsy. This approach is termed “incidence density control sampling.” When properly designed, such optimizations lead to the determination of odds ratios that are accurate reflections of the true relative risk of having prostate cancer, as either determined at initial or after repeat negative biopsy (Figure 2) [5, 31]. Several of the factors listed in Table 1, when measured in negative biopsies, have been shown to predict the presence of cancer at prostatectomy (e.g., [32, 33]), indicating that all such markers have this diagnostic potential.

### 2.2. Prostate Field Cancerization and Prognosis

The time of biopsy conceptually represents an ideal presurgical setting for clinical decision making for a patient at risk of having prostate cancer. However, even if the diagnosis of cancer can be achieved with high accuracy, a second related and as yet unresolved problem emerges at this point, that is, prognosis. While prognosis of progression is particularly powerful after surgical intervention, when the full extent of multifocality, distribution of grade, clinical staging (e.g., tumor volume), and analysis of surgical margins can be thoroughly performed, such assessment is much less accurate and often impossible in biopsies. Nevertheless, there is a growing consent among urologists that the detection of cancers in biopsies must be accompanied by the ability to determine the course of disease [16, 25]. Such conclusions are strongly supported by the large randomized trials of prostate cancer screening using the serum PSA test, that is, the European Randomized Study of Screening for Prostate Cancer (ERSPC) and the American Prostate, Lung, Colorectal, and Ovarian (PLCO) Cancer Screening Trial. Although the primary focus of these studies was to study the association between screening and mortality, they also showed that the accompanying increased rate of cancer detection (70% and 22% higher in the ERSPC and PLCO, resp.) did not substantially improve mortality rates [89, 90]. These results show that diagnosis and prognosis are closely interrelated issues and ideally, should not be addressed separately. In fact, it is now recognized that increasing detection of cancer in biopsies without the ability of being able to discriminate between aggressive and indolent disease can lead to the clinical overtreatment of patients who may remain asymptomatic throughout their lifetimes. Such patients may experience unnecessary treatment at a high cost-to-benefit ratio, including a lower quality of life, and would benefit more from active surveillance programs [91] (further discussed in Section 2.3). Although often problematic and controversial, staging and grading information for prognosis is routinely assessed in biopsy tissue. For example, all biopsy cores containing cancerous tissue are analyzed for their level of de-differentiation by assigning a Gleason grade. In addition, parameters such as the number of cores affected by cancer, and within a single core, the percentage of tissue affected by cancer as well as the location (especially at the apex for saturation biopsies) are used to predict tumor focality, size, and extracapsular extension which are classical staging parameters [16, 25]. However, the assessment of these parameters in biopsy tissues is frequently compromised by mischaracterization of the cancer. This is evidenced by the persistent problem of stage and grade migration after prostatectomy [25].

As with diagnosis, markers of field cancerization could be helpful to predict the course of disease. In fact, it is well accepted that the entire microenvironment, including stromal cells, extracellular matrix, and growth factors is a critical determinant of tumor cell behavior for most cancers [92]. In prostate cancer, the microenvironment has even been shown to be a controlling factor and key driver of tumor initiation and progression [93]. Importantly, the interaction between tumor cells and its associated stroma not only pertains to the tumor area itself, but extends to areas surrounding the tumor at an increased distance. This implies that characteristic markers of tumor adjacent tissues have predictive value in determining tumor initiation or progression. Such markers could be highly informative in both initially negative and positive biopsies (Figure 2). In negative cases, even after repeat biopsies, these markers could indicate whether adenocarcinoma develops subsequently. The value of these markers in initially positive biopsies from men with low-risk cancer is discussed in the next section in the context of active surveillance.

### 2.3. Prostate Field Cancerization and Active Surveillance

One option for the management of low-grade (Gleason score of ≤6) and low-stage (T1c or T2a) prostate cancer with PSA levels of ≤10 ng/mL is to delay or forego more aggressive treatment unless evidence of an increased risk of disease progression exists. This approach is called active surveillance and aims at avoiding the substantial side effects that accompany radical prostatectomy and radiation therapy, such as incontinence, impotence, and bowel dysfunction [89–91]. Active surveillance includes monitoring the patient's cancer with PSA tests and digital rectal examinations every 3–6 months, and performing prostate biopsies every 12–24 months. The optimal strategy for the latter seems to be the “staging saturation biopsy” approach to monitor the possibility of pathological upgrading and clinical upstaging [25, 28].

Conceptually, the statements made earlier about the potential of markers of field cancerization to indicate a possible progression towards increased malignancy apply here, too. It can be easily acknowledged that the longer-term monitoring approach which is at the heart of active surveillance is ideal to determine indicative and clinically meaningful changes over time (Figure 2). Regardless of whether such molecular changes in histologically intact tissues indicate progressive instability towards further cancer development, or whether they reflect the influence of a new or already existing and progressing tumor, a marker that can be assessed dynamically and quantitatively over time with high resolution would be of great value. Lastly, because saturation biopsy covers a larger area of the prostate gland, the concerted information from several biopsy cores can be used to identify possible “geographical patterns” of molecular alterations and their change over time. Since research on prostate field cancerization is new, it has not been applied to specific clinical scenarios such as active surveillance. Specific examples of such markers in this particular setting are thus missing from the literature. However, the feasibility for the prognostic potential of markers of field cancerization is greatly supported by the fact that several of the factors listed in Table 1 have been shown (partly in biopsies) to correlate with adverse patient outcome, such as biochemical (PSA) recurrence after radical prostatectomy [48, 65].

### 2.4. Prostate Field Cancerization and Preoperative Assessment of Positive Margins

A positive surgical margin is defined as tumor cells touching the inked edge of the specimen. This finding is reported in approximately 25% of cases. Positive margins are one of the main determinants of biochemical (PSA) relapse and are associated with a doubled or tripled risk of failure, depending on their number and location at the inked edge [94]. Ideal prognostication of margin status would entail preoperative and highly informative biopsies to predict the risk of a positive surgical margin and as a consequence, an extraprostatic extension at radical prostatectomy. In fact, preoperative knowledge about margin status, combined with other indicators of aggressiveness determined at the time of biopsy could greatly influence the choice of further intervention. To explore this possibility, the deliberate positioning of biopsy cores for tissue removal at the apex or the base, and their association with positive margins at the time of prostatectomy were previously investigated [95–98]. Interestingly, these investigations tend to be inconclusive with different studies reporting different outcomes. When individual core apical biopsies containing cancer were queried for their predictive value of positive surgical margin status and tumor involvement at the apex, it was less than 30% [97]. Similar results were reported when the incidences of a positive margin at both the apex and the base of the gland were analyzed for their association with the detection of positive or negative apical or basal biopsies [95]. In contrast, other studies determined the independent prognostic capability of positive preoperative apical biopsies for predicting positive surgical margins at the apex and reported positive predictive values of >70% [96]. Yet another study [98] reported conflicting results between apical and basal positive biopsies as predictors of positive surgical margins and extraprostatic extension. In this study, a positive biopsy at the base, but not at the apex, was predictive of a positive surgical margin and extraprostatic tumor involvement. Collectively, these studies show that the clinical value of preoperative analysis of apically and basally positioned biopsy needle cores to predict margin status remains inconclusive.

If markers of field cancerization are indicators of increased disease status, or indicators of extraprostatic cancer presence, it can be acknowledged that they could contribute to a more molecular and more refined interpretation of apical and basal biopsy material. Because research on prostate field cancerization is relatively new, examples specific to the assessment of positive margins are missing from the literature. However, the feasibility of such investigations has been announced [40], and given the importance of the zonal origin of biopsy cores taken from geographically distinct areas of the gland [99], it can be inferred from the studies on a variety of factors listed in Table 1.

### 2.5. Prostate Field Cancerization and Neo-Adjuvant Therapies

**Figure 3:**
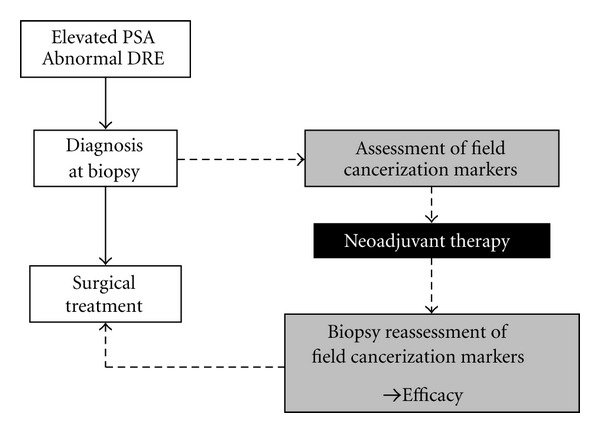
Study design to investigate the predictive potential of markers of field cancerization for pre-surgical neo-adjuvant therapies (black box), including the testing of novel agents in phase II clinical studies. Instead of applying surgical treatment with curative intent as a consequence of diagnosis of cancer at biopsy (white boxes), markers of field cancerization are assessed at the time of biopsy and after neo-adjuvant therapy (grey boxes) to determine its efficacy.

Neo-adjuvant intervention for prostate tumors is mostly indicated for high risk but localized organ-confined cancer. It can be applied as a monotherapeutic or multimodality approach and can entail radiotherapy, androgen ablation therapy, and chemotherapy [100]. The major goal of pre-surgical neo-adjuvant approaches is to improve the long-term outcome of subsequent prostatectomy with curative intent, as has been shown for all of the modalities listed above [101, 102]. An additional value of neo-adjuvant intervention is that it provides an opportunity for evaluating the activity and mechanism of action of neo-adjuvant new agents in correlative phase II clinical studies (Figure 3).

With respect to the latter, well-validated markers of field cancerization could function as surrogate endpoint indicators of therapeutic efficacy in biopsies removed before and after therapeutic intervention. Even when guided by ultrasound or other imaging techniques, it would be extremely difficult if not impossible to reliably resample the same premalignant tissue area in order to assess the effect of the therapeutic intervention. This is where markers of field cancerization could have a special advantage over markers that are specific for cancerous cells because they would be detected and quantitatively validated in structurally intact cells residing in histologically normal tissue associated with the lesion. In addition, assuming that the field is larger than the lesion itself (also see Figure 1), the exact position of the needle core biopsy would not matter as long as it is in the vicinity or area of the cancerous focus. Furthermore, as for most applications discussed in this paper, this possibility would be independent of whether the molecular alterations under investigation are precursors of the cancer or merely induced by its presence, as long as the field is representative of the tumor's response to the therapeutic intervention. Reports on specific examples of markers of field cancerization applied to the validation of neo-adjuvant therapies are missing from the literature, but are expected to increase once distinct molecular markers have been better characterized and validated.

### 2.6. Prostate Field Cancerization and Focal Therapy

Organ-preserving therapy is widely accepted for several types of tumors where the lesions are found to be small, well-differentiated, and confined. Organ sparing approaches include partial as opposed to radical resection, as well as focal treatment by cryotherapy, laser ablation, and high-intensity focused ultrasound. The obvious goal is to specifically destroy cancerous tissue areas while leaving the majority of the organ and the surrounding tissues unscathed and functional, and thereby avoiding substantial side effects and reduced quality of life accompanying radical prostatectomy [103, 104]. Because of the tendency to present with multifocal disease (see Section 1.2 above), focal therapy was not considered suitable for prostate cancer. However, several reasons have shifted this view towards a more favorable one. For example, the very essence of active surveillance (described in Section 2.3) is based on the notion that most lesions within a gland are indolent in nature and will not progress. Coupled with ever improving imaging and energy-delivery techniques, it is becoming increasingly feasible to detect and treat the primary (index) lesion. While the latter approach bears the risk of ignoring smaller yet potentially more aggressive lesions, it does nevertheless offer additional tumor control for patients choosing active surveillance. However, the benefits of focal intervention will have to be determined in large trials before this approach can be recommended in all men with low-risk progression prostate cancer.

**Figure 4:**
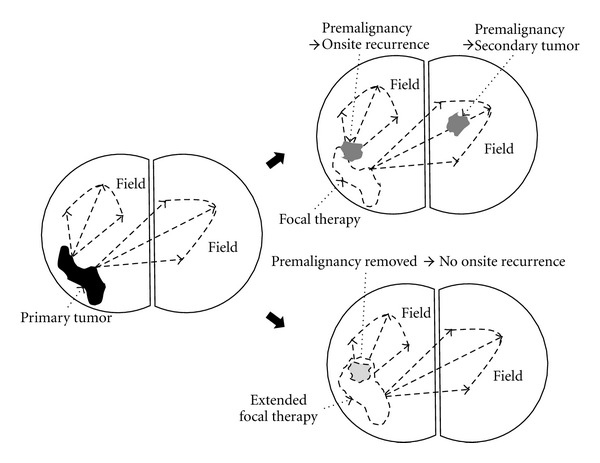
A primary tumor (black irregular shape) is associated with a complex field of molecular alterations in structurally intact cells in histologically normal adjacent tissue (arrows and dotted lines; left). After focal therapy to the primary tumor (white dashed lined irregular shape), premalignant areas (grey irregular shapes) may in time lead to onsite or secondary tumors within the remaining field (upper right). If focal therapy is extended to include the field, onsite recurrences could be avoided (lower right).

The biology of field cancerization conceptually opposes the use of focal therapy for the treatment of prostate cancer because the attempted eradication of histologically cancerous tissue would leave behind structurally intact yet molecularly altered and genetically compromised tissues that may contain cells with the potential to cause onsite recurrences and/or secondary tumors (Figure 4). However, at least three deviations from this view should be mentioned. First, if markers of field cancerization are able to discriminate with high accuracy histologically normal yet genetically compromised (i.e., field cancerized) from histologically and genetically intact (i.e., truly normal) tissues, the extent of the field associated with an individual lesion would become defined and could be included in the focal therapeutic approach (Figure 4). Second, if markers of field cancerization are predictive of progression (as discussed in Section 2.2), indolent lesions could be discriminated from more aggressive foci, thereby increasing the efficacy of focal therapy by guiding it towards foci with higher risk of progression. Lastly, if markers of field cancerization are merely indicators of the presence of cancer cells, as opposed to precursors of disease (as discussed in Section 1.1), they could be of value as predictive indicators for the efficacy of focal intervention (similar to their application described in Section 2.5).

## 3. Conclusions

Prostate cancer is an extensively heterogeneous disease with highly variable clinical outcome. The time of biopsy is an important milestone for a patient at risk of having cancer and progressing to a more advanced stage. Apart from confirmatory diagnosis, the information gained from biopsy cores is also crucial in determining further actions, especially the choice between active surveillance and more aggressive therapeutic interventions, including radical prostatectomy (Sections 2.1 to 2.4) [25, 105]. The procurement and analysis of biopsies also play a role during therapeutic intervention, such as in predicting and monitoring the efficacy of pre-surgical neo-adjuvant and focal therapy (Sections 2.5 and 2.6) [100, 104]. Therefore, the clinical management of prostate cancer leads to millions of biopsy cores that are removed by urologists and analyzed by pathologists each year in the USA [21, 24, 25], underscoring the importance of this type of material. Unfortunately, the analysis and interpretation of prostate biopsies is often complicated by limiting factors such as the number of cores affected by cancer, the amount of glandular tissue in an individual core, and the amount of tissue in general. In addition, there remains discordance, despite extended biopsy schemes, between the interpretation from biopsy material and information gained at prostatectomy, as evidenced by the frequency of upstaging and upgrading [25, 28].

Markers of field cancerization have the potential to overcome these important barriers. This is due primarily to the fact that molecular alterations that deviate from normalcy can be assessed in a larger target, defined as the tissue area adjacent to the tumor lesion, thus becoming more independent of the analysis of the lesion itself. However, key to such optimization is the choice of the proper controls for potentially field cancerized tissues, as the focus is shifted to what most investigators use as the control for the analysis of tumor tissues [20]. Tissues unrelated to cancer and similar conditions should be chosen and matched for age, body mass, and other factors to avoid detection of false positive molecular aberrations. This may be challenging due to intertissue heterogeneity and requires extensive characterization of the biomarker under investigation with respect to its association with cancer presence or development. The latter necessitates detailed knowledge about the nature and extent of the field associated with tumor foci. This knowledge, however, has just begun to be generated for men at risk of having or developing prostate cancer. Nevertheless, data in support of the potential of markers of field cancerization to help solve the “cancer biomarker problem,” as emphasized recently by leaders in the field [21, 106], is accumulating (Table 1) and warrants further research.

## Figures and Tables

**Table 1:** Molecular markers that are in accordance with the definition of prostate field cancerization reported in the literature^1^.

Type of molecular alteration^2^	Specific molecular marker	Reference^3^
Cytomorphological changes	Nuclear chromatin structure	[34]
	Nuclear chromatin structure	[35]
	Nuclear texture	[36]
Epigenetic changes	Methylation of GSTp1 and RAR*β*2	[37]^4^
	Methylation of GSTp1, RAR*β*2, and APC	[38]
	Methylation of RASSF1A	[39]
	Methylation of RAR*β*2, APC, and RASSF1A	[40]
	Methylation of HIN-1	[41]
Genomic DNA changes	Profile of infrared spectroscopy	[42]
	Profile of infrared spectroscopy	[43]
	Profile of infrared spectroscopy	[44]
	Telomere attrition	[45]
	Telomere attrition	[46]
	TMPRSS2-ERG fusion	[47]
	Content of DNA	[32]
	Telomere attrition	[48]
	Telomere attrition	[49]
	Telomere attrition	[50]^4^
Mitochondrial DNA changes	Mutation	[51]
	Deletion	[52]
	Deletion	[53]
Gene expression changes	Gene expression signature (671 genes)	[54]
	cDNA microarrays (12,625 probes)	[55]
	Noncoding RNA from PCA3 gene	[56]
	Panel of 8 genes	[57]
	Gene fusion transcript of TMPRSS2-ERG	[58]
	cDNA microarrays (37,123 probes)	[59]
	PSCA	[60]
Protein expression changes	PS2	[61]
	Androgen receptor	[62]^4^
	COX-2	[63]
	Androgen receptor	[64]^4^
	Phosphorylated Akt-1	[65]
	EPCA	[66]
	EPCA	[67]
	Calcium channel P2X7	[33]
	AMACR	[68]
	Ki67 and MCM-2	[69]
	Activated Akt	[70]
	EPCA	[71]
	AMACR	[72]
	*β*-catenin	[73]
	Androgen receptor	[74]^4^
	Expression of Akt	[75]
	PDGFR*β*	[76]^4^
	Phosporylated EGFR	[77]
	EGR-1 and IGF1R	[78]
	UDP-glucose dehydrogenase	[79]
	Prostate tumor overexpressed 1	[80]
	EGR-1 and FAS	[81]
NMR spectra	Metabolites	[82]

^1^Combined and updated from Nonn et al., 2009 [5] and Halin et al., 2011 [4].

^2^Additional markers of field cancerization that are not strictly molecular in nature, but rather represent cellular changes or adaptations to the presence of the tumor are cell morphological architecture [83], increased numbers of mast cells and macrophages [84–86], and enhanced microvessel density [87, 88].

^3^Within a type of molecular alteration group, the studies are reported in chronological order; references are original articles only; additional review articles are listed in the text.

^4^Reported in stromal cells.
